# Imaging of Foreign Body Reactions to Non-absorbable Sutures in Achilles Tendon Repair: Radiologic and Histopathologic Correlation in Two Cases

**DOI:** 10.7759/cureus.79224

**Published:** 2025-02-18

**Authors:** Juan Pablo Munoz, Julio Botello, Ignacio Espinoza, Ariel Valle, Sebastián Figueroa

**Affiliations:** 1 Skeletal Radiology, Clínica MEDS, Santiago, CHL; 2 Ankle Orthopedic Surgery, Clínica MEDS, Santiago, CHL

**Keywords:** achilles repair, foot & ankle surgery, foreign-body reaction, mri, ultrasound

## Abstract

Achilles tendon ruptures are commonly managed with surgical repair, often using non-absorbable sutures for their tensile strength. However, foreign body reactions have been increasingly reported, accounting for up to 2% of complications in some studies, compared to approximately 3% for wound dehiscence.

We present two cases of biopsy-proven foreign body reactions following percutaneous Achilles tendon repair with FiberWire® (Arthrex, Naples, Florida) sutures. Both patients developed localized swelling and erythema months after surgery, without any systemic signs of infection. Ultrasound and MRI revealed cystic-like lesions along the suture path, accompanied by inflammatory changes. Surgical debridement and suture removal led to complete resolution of symptoms, with corresponding normalization on follow-up imaging.

Histopathology confirmed a foreign body reaction with giant cells and reactive fibroblast deposition. These cases emphasize the importance of recognizing foreign body reactions as a potential mimic of infection or rerupture, guiding appropriate management.

## Introduction

Achilles tendon injuries are among the most common tendon ruptures, with an incidence of approximately 2.1 per 100,000 person-years in the United States, a figure that continues to rise in larger studies [1]. Complete ruptures are managed with both non-surgical and surgical treatments, with the latter yielding lower rerupture rates [2]. Non-absorbable sutures are more commonly used than absorbable sutures for repairs [3]. Complications following repair include rerupture, infection, sural nerve injury, deep vein thrombosis, and wound dehiscence [4]. After tenorrhaphy, healing occurs in three stages, inflammation, repair, and remodeling, progressing centripetally. The inflammatory stage lasts ~24 hours, with white blood cell accumulation, angiogenesis, and tenocyte proliferation initiating collagen synthesis. Collagen production peaks in the reparative stage over the following weeks. The remodeling stage begins at six weeks, lasting up to a year, as repair tissue transitions into scar-like tendon tissue [5]. 

On MRI, acute scar tissue within the tendon gap appears T2-hyperintense in the early postoperative weeks. The gap size may be overestimated, reflecting both physical separation and tendon-end remodeling. By seven to nine weeks, granulation tissue fills the gap, though mature fibrous tissue remains minimal. Around 12 weeks, the gap resolves, replaced by T1- and T2-hypointense fibrotic scar tissue [6]. There is increasing evidence that non-absorbable sutures can cause foreign body reactions, sometimes necessitating open removal [4,7,8]. These reactions are characterized by persistent inflammation at the implant interface, progressing to granulation tissue formation. This process involves macrophages, fibroblasts, neovascularization, and the eventual development of a fibrous capsule separated from the implant by giant cells and macrophages [9]. While foreign body reactions to both absorbable and non-absorbable sutures have been documented [7], their imaging characteristics in Achilles tendon repair remain underexplored and are primarily described in case reports [8,10,11]. Esenyel et al. compared three non-absorbable sutures, reporting that FiberWire® (Arthrex, Naples, Florida) caused a wider inflammation zone than Ethibond® (Ethicon, Somerville, New Jersey) but a narrower one than polypropylene at six weeks [12]. FiberWire® combines polyethylene core fibers with a braided polyester jacket, offering superior tensile strength and handling properties. Here, we present two cases of Achilles tendon repair performed through a percutaneous approach using FiberWire®, with subsequent biopsy-proven foreign body reactions. Our report includes multimodal imaging findings, pre- and post-suture removal, illustrating the characteristics of this underrecognized complication.

## Case presentation

Case 1

**Figure 1:**
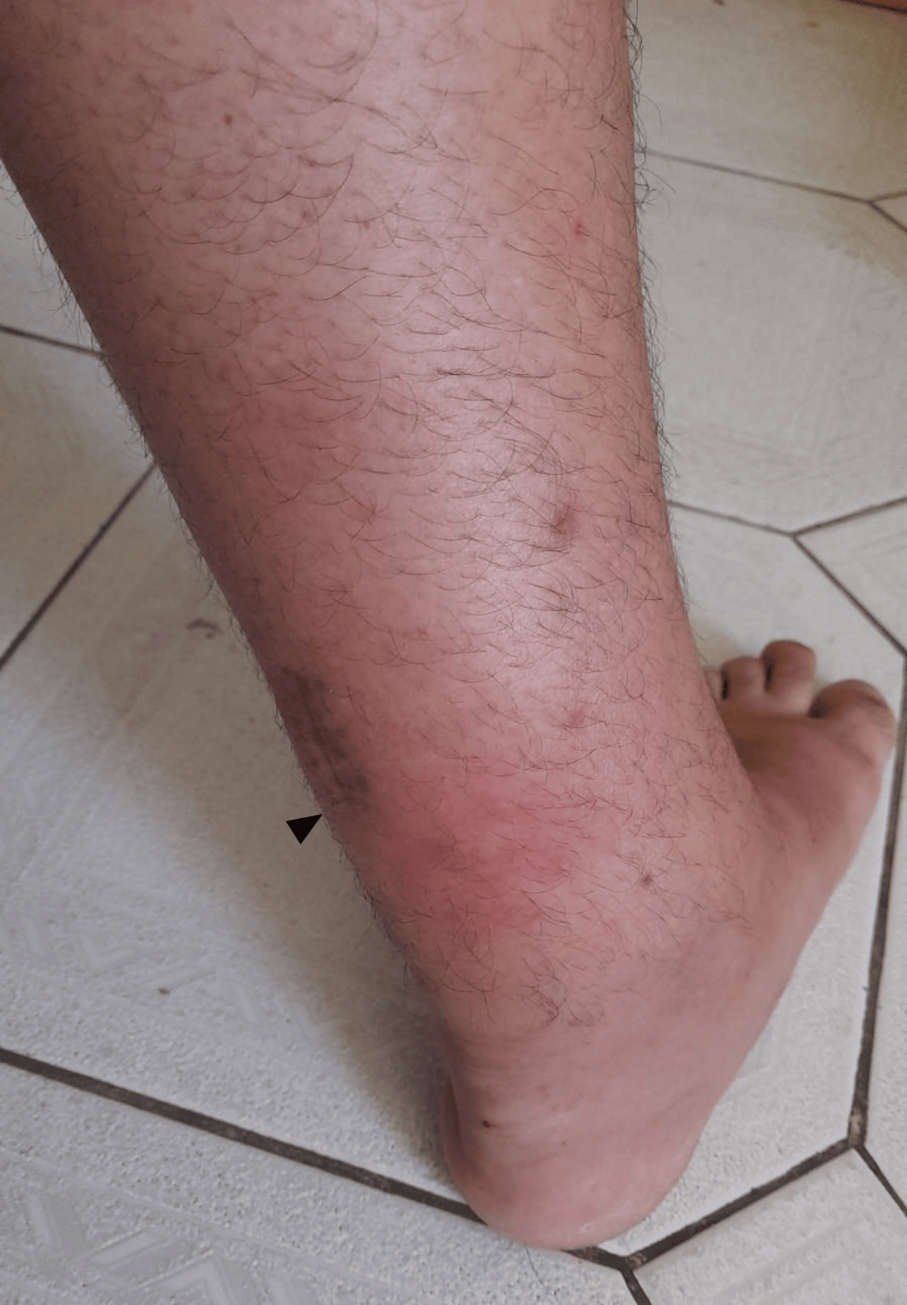
Posterior leg (Patient 1) Posterior leg showing skin erythema (black arrowhead) around the suture entry points.

A 36-year-old male, engaged in regular recreational physical activity (but not high-performance training), underwent percutaneous Achilles tendon repair for a complete rupture using three FiberWire® sutures. His postoperative recovery was uneventful, and he returned to baseline activities. Our institutional rehabilitation protocol for Achilles tendon repair aligns with internationally recognized milestones, emphasizing early functional rehabilitation, progressive weight-bearing, and range of motion exercises. While no universally accepted protocol exists, systematic reviews highlight significant heterogeneity and a lack of consensus in the literature [13]. The American Orthopaedic Foot & Ankle Society (AOFAS) does not endorse a standardized protocol but supports early mobilization, a principle reflected in our approach [14]. Our five-phase rehabilitation protocol includes Phase 1 (weeks 0-2) - Controlled Ankle Motion (CAM) boot with early weight-bearing; Phase 2 (weeks 2-6) - progressive weight-bearing, ROM, and isometric strengthening; Phase 3 (weeks 6-12) - full weight-bearing and seated heel raises; Phase 4 (weeks 12-24) - boot removal, dynamic strengthening, and low-impact activities; Phase 5 (months 6-12) - gradual return to sports. At four months post-surgery (Phase 4), the patient developed localized ankle swelling and erythema at the suture entry points, findings inconsistent with expected wound healing and raising suspicion of late-onset infection (Figure 1).

However, there were no systemic signs of infection. Laboratory tests, including white blood cell count, erythrocyte sedimentation rate, and C-reactive protein, were within normal limits (Table 1).

**Table 1:** Relevant laboratory findings (Patient 1)

Parameters	Value	Reference range	
White blood cell count	6.3×10³/µL	4.5-11×10³/µL	
Erythrocyte sedimentation rate	2 mm/hr	1-14 mm/hr	
C-reactive protein	<0.06 mg/dL	<0.5 mg/dL	

**Figure 2:**
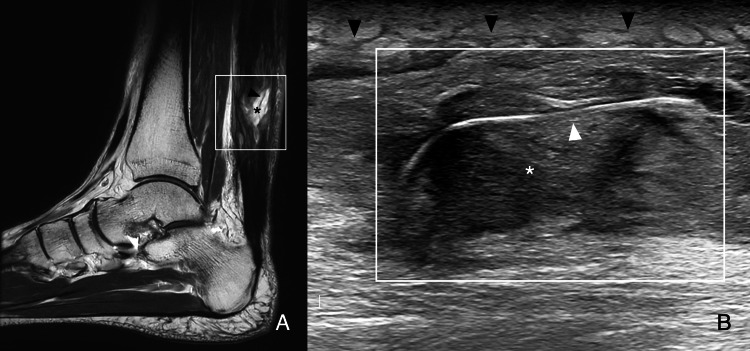
MRI and ultrasound (Patient 1) A) Sagittal T2-weighted image of the ankle shows a cystic-like lesion (black asterisk) aligned with the suture path (black arrowhead). B) Ultrasound B-mode, long axis: sutures (white arrowhead) embedded in heterogeneous material (white asterisk) with subcutaneous inflammatory changes (black arrowheads). Color Doppler also revealed hyperemia (not shown). The white box in A indicates the scanned region shown in B.

MRI revealed cyst-like midsubstance lesions with a lobulated appearance and high signal on T2-weighted and proton density fat-saturated (PDFS) images, consistent with granulation tissue in contact with the sutures, which appeared as linear hypointense lines on T2-weighted images, along with inflammatory peritendinous changes (Figure 2A). Ultrasound revealed heterogeneous midsubstance material, both anechoic and hypoechoic, adjacent to the sutures, appearing as a linear hyperechoic focus on B-mode, along with increased peritendinous echogenicity and intra- and peritendinous color Doppler hyperemia (Figure 2B).

He underwent open surgical suture removal and debridement for suspected foreign body reaction, without prior antibiotic treatment. Under spinal anesthesia, the patient was positioned prone. Using ultrasound guidance, a skin mark was made over the granulation tissue surrounding the sutures, aligned with the previous incision scar. The sutures and surrounding granulomatous tissue were excised via a mini-open approach. After excision, the remaining tendon gap was repaired with non-absorbable sutures. No graft or tendon transfer was required.

**Figure 3:**
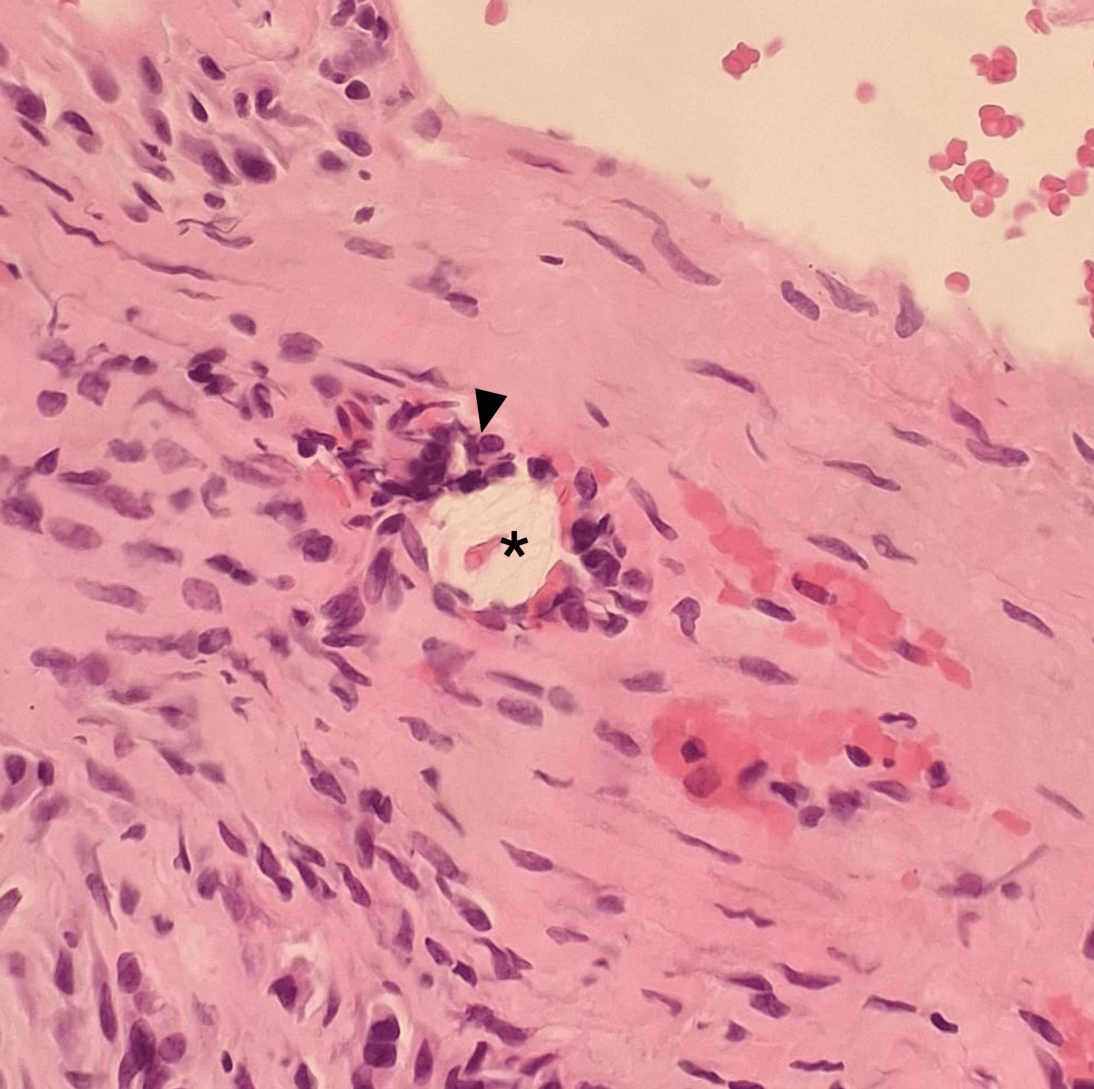
Histology (Patient 1) Hematoxylin and eosin stain (200×) reveal hyalinized tissue with reactive fibroblasts and foreign body giant cells (black arrowhead) surrounding the suture material (black asterisk).

The intraoperative findings primarily showed granulation tissue, with small amounts of fluid in contact with the sutures. Microbiological analysis revealed no infection. Histological findings included aggregates of giant cells surrounding suture material, fibrinoid degeneration, hyaline tissue, reactive fibroblasts, and mononuclear infiltrates (Figure 3).

**Figure 4:**
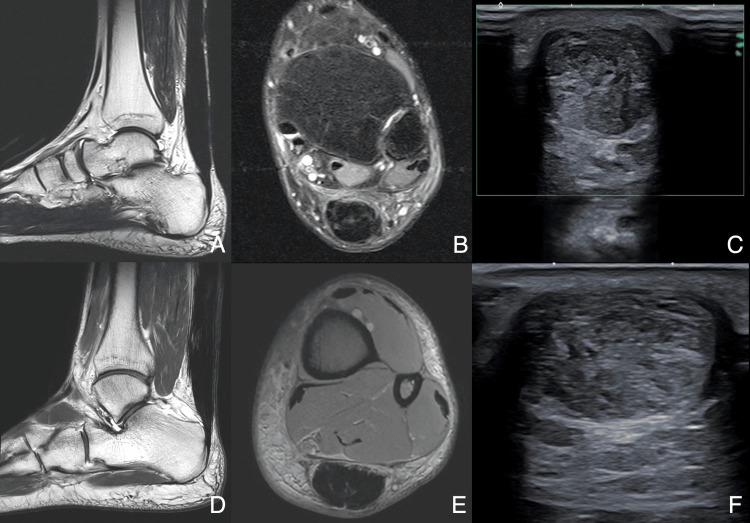
Post-suture removal imaging of the two patients Postoperative Patient 1 (three months: A-C) and Patient 2 (four months: D-F). (A, D) Sagittal T2-weighted images show complete resolution of the cystic-like lesion. (B, E) Axial PDFS confirms the resolution of the cystic-like lesion and soft tissue edema. (C, F) Ultrasound: (C) B-mode power Doppler, transverse axis, shows the absence of hyperemia within the Doppler interrogation box (green). (F) B-mode, transverse axis, shows normal postoperative tendon thickening. PDFS, proton density fat-saturated

**Figure 5:**
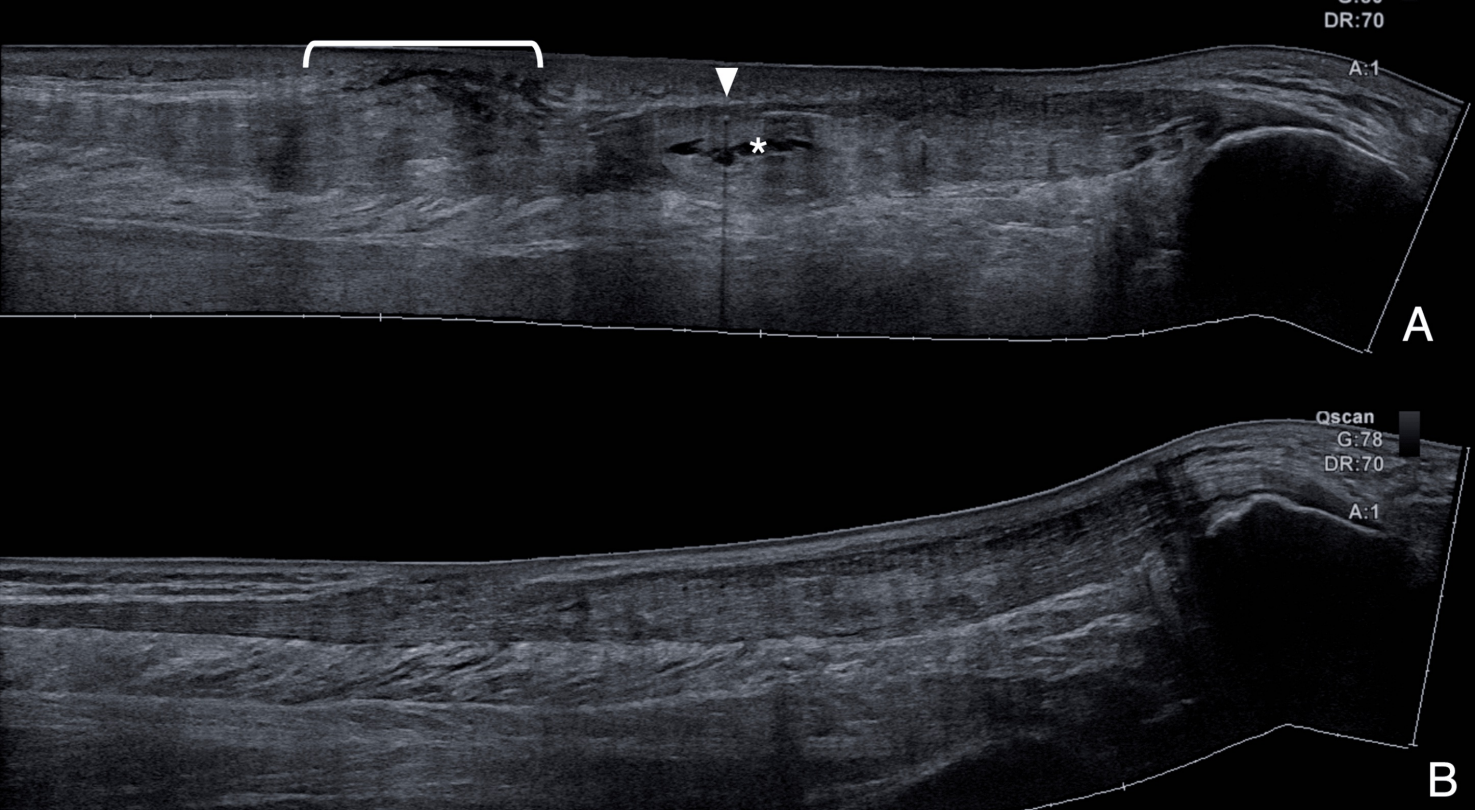
Ultrasound showing restoration of normal post-tenorrhaphy status (Patient 1) A) Pre-suture removal B-mode ultrasound extended view reveals a cystic-like lesion (white asterisk) adjacent to the suture path (white arrowhead) and subcutaneous inflammatory changes (white bracket). B) Post-suture removal B-mode ultrasound extended view shows restoration of normal post-tenorrhaphy status.

At three months post-debridement, a follow-up MRI demonstrated complete resolution of cystic-like lesions and peritendinous inflammatory changes. Ultrasound revealed restored postoperative tissue characteristics, including appropriate echogenicity and thickness, with no Doppler hyperemia or inflammatory signs (Figure 4 and Figure 5).

Case 2

A 43-year-old male, also engaged in regular recreational physical activity, underwent percutaneous Achilles tendon repair for a complete rupture using FiberWire® sutures. His postoperative course was uneventful, and he resumed baseline activities.

At five months post-surgery (Phase 4), the patient developed localized ankle swelling and erythema, resembling the presentation in Patient 1 and initially raising suspicion of late-onset infection. However, laboratory tests were within normal limits (Table 2).

**Table 2:** Relevant laboratory findings (Patient 2)

Parameters	Value	Reference range	
White blood cell count	7.1×10³/µL	4.5-11×10³/µL	
Erythrocyte sedimentation rate	5 mm/hr	1-14 mm/hr	
C-reactive protein	0.08 mg/dL	<0.5 mg/dL	

**Figure 6:**
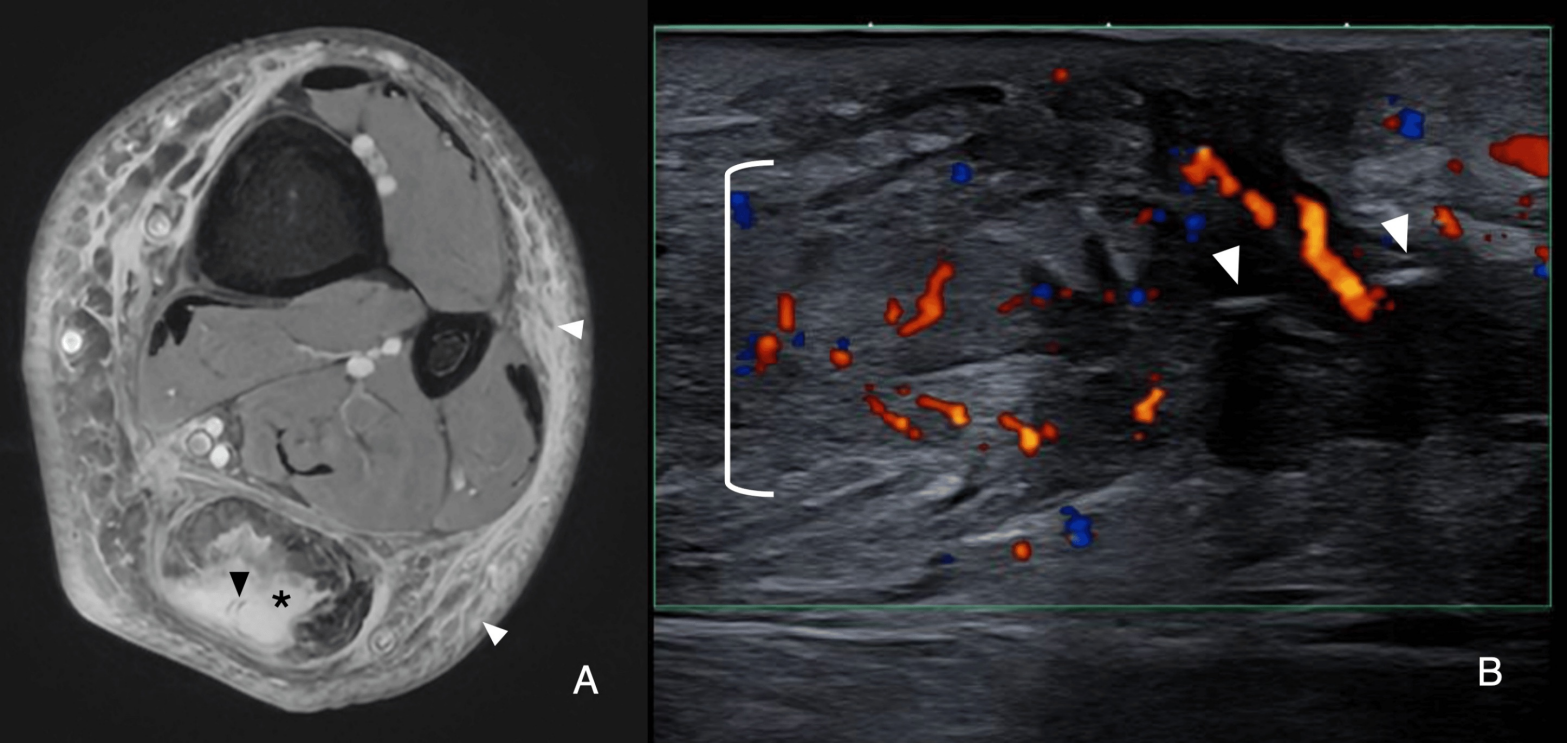
MRI and ultrasound (Patient 2) A) Axial PDFS reveals a cystic-like lesion (black asterisk) surrounding the sutures (black arrowhead), with associated soft tissue edema (white arrowheads). B) Long-axis color Doppler shows Doppler hyperemia (white bracket) surrounding the sutures (white arrowheads). PDFS, proton density fat-saturated

Imaging findings mirrored those of the first case. MRI showed cyst-like midsubstance lesions with high signal on T2-weighted and PDFS images surrounding the sutures, appearing as linear hypointense lines, along with inflammatory peritendinous changes (Figure 6A). Ultrasound revealed hypoechoic, heterogeneous material adjacent to the sutures, depicted as a linear hyperechoic focus on B-mode, increased peritendinous echogenicity, and color Doppler hyperemia (Figure 6B).

He underwent open surgical suture removal and debridement without prior antibiotic treatment. Similarly, Patient 2 underwent the same procedure. Ultrasound guidance was used to mark the skin over the granulation tissue surrounding the sutures, corresponding to the previous incision. The excision of the sutures and granulomatous tissue was performed using a mini-open approach, followed by repair of the tendon gap with non-absorbable sutures, with no need for grafts or tendon transfers.

**Figure 7:**
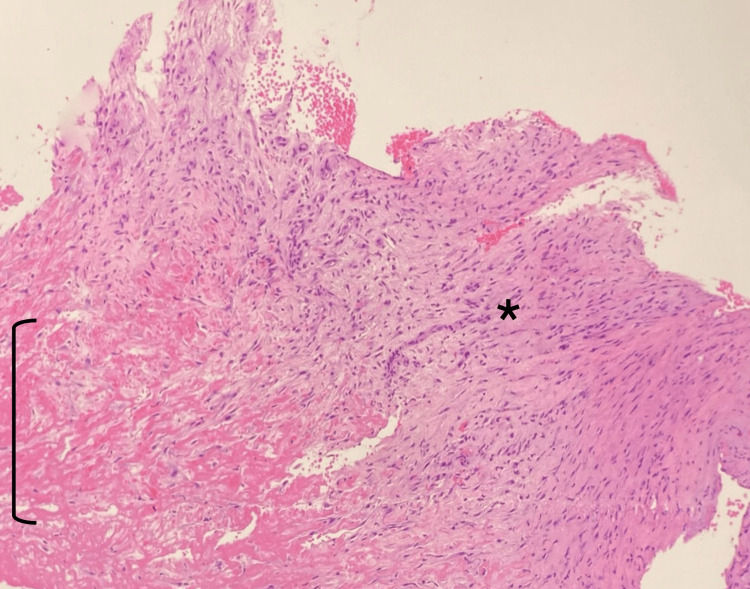
Histology (Patient 2) Hematoxylin and eosin stain (40x) reveal connective tissue with fibrinoid degeneration (black bracket) and fibrous scar zone with reactive fibroblasts (black asterisk).

Similar to Patient 1, the intraoperative findings primarily revealed granulation tissue in contact with the sutures. Microbiological analysis ruled out infection. Histological findings were similar to Case 1, showing fibrinoid degeneration, hyaline tissue, reactive fibroblasts, and mononuclear infiltrate (Figure 7).

At four months post-debridement, follow-up MRI showed complete resolution of cystic-like lesions and inflammatory changes. Ultrasound confirmed normal postoperative tissue characteristics, with appropriate echogenicity and thickness, and no inflammatory signs (Figure 5).

## Discussion

Management of Achilles tendon ruptures involves operative and non-operative approaches. Surgical repair reduces the risk of rerupture by 73% compared to non-operative treatment but carries higher complication rates, including infection, sural neuritis, thrombosis, and adhesions [2]. Open and percutaneous techniques, initially believed to differ in tensile strength and rerupture rates, have shown comparable outcomes in recent studies [4,15]. High T2 signal midsubstance lesions on MRI are common during normal healing after tenorraphy, resolving in a centripetal pattern [16]. Reported resolution times vary, with some studies showing resolution in most patients by three months, while others describe persistent small hyperintense areas at 19±11 months in 30% of cases [6,17]. This overlap between normal healing and pathology necessitates careful interpretation of imaging findings. High water content lesions on ultrasound and MRI can resemble cystic or cystic-like entities, including synovial cysts, bursae, postsurgical collections, and abscesses. They appear hypo- or anechoic with deep acoustic enhancement on grayscale ultrasound. Doppler imaging helps differentiate them by detecting vascularization. On MRI, they are hypointense on T1 and hyperintense on T2 due to prolonged T2 relaxation [18]. Foreign body reactions to non-absorbable sutures are a recognized complication of surgical repair, characterized histologically by chronic inflammation, granulation tissue formation, and fibrosis [9]. While their histopathological features are well-documented, their imaging characteristics in Achilles tendon repair remain underexplored. Our report presents multimodal imaging findings (ultrasound and MRI) of biopsy-proven foreign body reactions to FiberWire® sutures and is the first to include post-suture removal imaging. Ultrasound demonstrated midsubstance hypoechoic material in contact with the sutures aligned with the tendon axis, increased subcutaneous echogenicity, and Doppler hyperemia. MRI showed high-signal cystic-like lesions on fluid-sensitive sequences, identifiable sutures within the fluid, and inflammatory subcutaneous changes. Notably, the MRI detected cystic-like lesions that did not always correspond to anechoic foci on ultrasound. Instead, hypoechoic heterogeneous material, likely granulation tissue, frequently surrounded the sutures. Subcutaneous inflammation, underemphasized in prior reports, was prominent in our cases and may clinically mimic infection. In the largest series by Kim et al., eight patients with Ethibond® suture reactions were evaluated an average of 25 months post-tenorrhaphy, with similar intratendinous imaging findings [8]. While their analysis included preoperative imaging, post-suture removal findings were not reported. In contrast, our cases involved FiberWire® sutures, presented five months post-surgery, and included both pre- and post-suture removal imaging. Additionally, unlike prior reports that noted sinus tract formation, this feature was absent in our patients [7]. The absence of previous reports on imaging after suture removal highlights the contribution of our two cases in assessing the resolution of midsubstance granulation tissue, the rapid progression to scar formation, and the restoration of the expected post-tenorrhaphy status. These findings provide valuable insight for physicians managing this underrecognized entity. Our findings align with prior observations but provide additional insights into subcutaneous inflammatory changes and the multimodal imaging appearance of suture reactions. Recognizing these imaging features facilitates timely diagnosis and appropriate management of this complication.

## Conclusions

Foreign body reaction following Achilles tendon repair is an increasingly recognized phenomenon both clinically and radiologically, more frequently reported with non-absorbable sutures. In the absence of elevated inflammatory markers, midsubstance cystic-like lesions following the suture path, along with surrounding soft tissue inflammation and possible sinus tract formation, suggest a foreign body reaction. While these imaging findings are not pathognomonic, they aid in detection and differentiation from other postoperative complications. Surgical removal of the sutures may lead to complete resolution of both imaging findings and clinical symptoms.
